# Possible mechanisms involved in the effect of the subchronic administration of rosuvastatin on endothelial function in rats with metabolic syndrome

**DOI:** 10.1590/1414-431X20199304

**Published:** 2020-02-10

**Authors:** J. Lozano-Cuenca, I. Valencia-Hernández, O.A. López-Canales, H. Flores-Herrera, R.M. López-Mayorga, E.F. Castillo-Henkel, J.S. López-Canales

**Affiliations:** 1 Department of Physiology and Cell Development, National Institute of Perinatology, Mexico City, Mexico; 2 Section of Postgraduate Studies and Investigation, Higher School of Medicine, National Polytechnic Institute, Mexico City, Mexico; 3 Department of Immuno-Biochemistry, National Institute of Perinatology, Mexico City, Mexico

**Keywords:** Rosuvastatin, Metabolic syndrome, Rat aorta, Vasorelaxation, NO, K^+^ channel

## Abstract

Metabolic syndrome is a multifaceted condition associated with a greater risk of various disorders (e.g., diabetes and heart disease). In a rat model of metabolic syndrome, an acute *in vitro* application of rosuvastatin causes relaxation of aortic rings. Since the outcome of a subchronic rosuvastatin treatment is unknown, the present study explored its effect on acetylcholine-induced vasorelaxation of aortic rings from rats with metabolic syndrome. Animals were submitted to a 16-week treatment, including a standard diet, a cafeteria-style diet (CAF-diet), or a CAF-diet with daily rosuvastatin treatment (10 mg/kg). After confirming the development of metabolic syndrome in rats, aortic segments were extracted from these animals (those treated with rosuvastatin and untreated) and the acetylcholine-induced relaxant effect on the corresponding rings was evaluated. Concentration-response curves were constructed for this effect in the presence/absence of L-NAME, ODQ, KT 5823, 4-aminopyridine (4-AP), tetraethylammonium (TEA), apamin plus charybdotoxin, glibenclamide, indomethacin, clotrimazole, and cycloheximide pretreatment. Compared to rings from control rats, acetylcholine-induced vasorelaxation decreased in rings from animals with metabolic syndrome, and was maintained at a normal level in animals with metabolic syndrome plus rosuvastatin treatment. The effect of rosuvastatin was inhibited by L-NAME, ODQ, KT 5823, TEA, apamin plus charybdotoxin, but unaffected by 4-AP, glibenclamide, indomethacin, clotrimazole, or cycloheximide. In conclusion, the subchronic administration of rosuvastatin to rats with metabolic syndrome improved the acetylcholine-induced relaxant response, involving stimulation of the NO/cGMP/PKG/Ca^2+^-activated K^+^ channel pathway.

## Introduction

Metabolic syndrome is a multifaceted condition characterized by a group of abnormalities such as abdominal obesity, hypertension, dyslipidemia, and high blood pressure (1). The presence of metabolic syndrome is associated with a significantly greater risk of diverse disorders, including diabetes, coronary heart disease, cerebrovascular disease, hypertension, and endothelial dysfunction.

Statins able to inhibit 3-hydroxy-3-methylglutaryl coenzyme A (HMG-CoA) reductase are among the drugs used to treat metabolic syndrome. One of the pleiotropic cholesterol-lowering effects attributed to statins is the improvement in endothelial function resulting from an increase in the expression and activity of endothelial nitric oxide synthase (NOS) (2,3). There is evidence of mechanisms involved in the vasorelaxation produced by an acute *in vitro* application of rosuvastatin on aortic rings of rats fed with a cafeteria-style diet (CAF-diet: a model of metabolic syndrome and endothelial dysfunction) (4). However, regarding a subchronic treatment with rosuvastatin, the mechanisms involved in the improvement of endothelial function in rats subchronically fed with a CAF-diet, remain unknown.

The present study set out to analyze in aortic rings of rats subjected to the CAF-diet the possible mechanisms responsible for an improved vasodilator response caused by acetylcholine after subchronic treatment with rosuvastatin. To explore the mechanism of action of the relaxant response to acetylcholine, an evaluation was made of the effect on this response produced by various compounds: 10^−5^ M N_ω_-nitro-L-arginine methyl ester (L-NAME; a direct inhibitor of NO synthase), 10^−7^ M 1H-(1,2,4) oxadiazolo[4,3-a] quinoxalin-1-one (ODQ; an inhibitor of soluble guanylyl cyclase enzyme), 10^−6^ M (9S,10R,12R)-2,3,9,10,11,12-hexahydro-10-methoxy-2,9-dimethyl-1-oxo-9,12-epoxy-1H-diindolo [1,2,3fg:3’,2’,1’-kl] pyrrolo[3,4-i] (1,6) benzodiazocine-10-carboxylic acid, methyl ester (KT 5823; an inhibitor of protein kinase G), 10^−3^ M 4-aminopyridine (4-AP; a voltage-activated K^+^ channel blocker), 10^−2^ M tetraethylammonium (TEA; a Ca^2+^-activated K^+^ channel blocker and non-specific voltage-activated K^+^ channel blocker), 10^−7^ M apamin plus 10^−7^ M charybdotoxin (blockers of small- and large-conductance Ca^2+^-activated K^+^ channels, respectively), 10^−7^ M glibenclamide (an ATP sensitive K^+^ channel blocker), 10^−5^ M indomethacin (a prostaglandin synthesis inhibitor), 10^−5^ M clotrimazole (a cytochrome P450 inhibitor), and 10^−5^ M cycloheximide (a general protein synthesis inhibitor).

## Material and Methods

### Animals

Seventy-two male Wistar rats were purchased from the Escuela Superior de Medicina (Mexico), housed in plastic cages in a special temperature-controlled room (22±2°C, 50% humidity), and kept on a 12-h light/dark cycle (lights on at 7 am). They were randomly distributed into three groups that received for 16 weeks: i) a standard diet (Rat chow 5012, Pet Foods Home, Mexico); ii) a CAF-diet containing 33% ground commercial rat chow, 33% full-fat sweetened condensed milk (Nestlé), 7% sucrose, and 27% water (n=36), as reported by López-Canales et al. (4); or iii) a CAF-diet plus rosuvastatin administered orally (10 mg/kg) once per day. All animals had free access to drinking water throughout the experiments. The study was approved by the Animal Care Committee of Escuela Superior de Medicina (Mexico), and was in agreement with the 1986 Animals (Scientific Procedures) Act of the Parliament of the United Kingdom (http://www.legislation.gov.uk/ukpga/1986/14/contents).

### Measurement of metabolic parameters

Serum insulin concentrations were determined with the rat insulin Elisa kit 90010 (Crystal Chemical Co. USA). The blood glucose concentrations were evaluated using an Accu-Check Active auto-analyzer (Roche, Germany). Total cholesterol and triglyceride levels were determined in blood samples after 12 h fasting (5) with the Accutrend Plus auto-analyzer (Roche). According to the manufacturer, this instrument has an intra-assay precision of 3.7% for total cholesterol and 3.4% for triglycerides. Using controls, we calibrated the intra-assay precision as 5% for total cholesterol and 2.4% for triglycerides. Blood pressure was evaluated with the CODA^®^ mouse rat tail-cuff system, a noninvasive small animal blood pressure monitoring system (Kent Scientific Corp., USA).

### Preparation of aortic rings to analyze the vascular effect of rosuvastatin

Animals were euthanized by decapitation and the aortas were immediately excised and placed in cold buffer, then cleaned and freed from surrounding connective tissue. The isolated arteries were cut into rings (4–5 mm long) and put into 10-mL tissue chambers filled with Krebs-Henseleit bicarbonate buffer (1.18×10^−1^ M NaCl, 4.7×10^−3^ M KCl, 1.2×10^−3^ M KH_2_PO_4_, 1.2×10^−3^ M MgSO_4_·7H_2_O, 2.5×10^−3^ M CaCl_2_·2H_2_O, 2.5×10^−2^ M NaHCO_3_, 1.17×10^−2^ M dextrose, and 2.6×10^-5^ M calcium disodium EDTA). Tissue baths, maintained at 37°C and pH 7.4, were bubbled with a mixture of 95% O_2_ and 5% CO_2_.

Aortic rings were mounted onto two stainless steel hooks to record the isometric tension. One was fixed to the bottom of the chamber and the other to a BIOPAC TSD125C-50g force transducer connected to a BIOPAC MP100A-CE data acquisition system (BIOPAC Systems, Inc., USA). The optimal tension selected from preliminary experiments was that which had the greatest response to phenylephrine (10^−6^ M). The rings were given about 2.0 g (100%) of initial tension and allowed to equilibrate for 2 h. Thirty minutes after setting up the organ bath, tissues were first contracted with 10^−6^ M phenylephrine to test their contractile responses. These were then rinsed three times with Krebs solution to restore tension to precontraction levels.

Endothelial integrity was pharmacologically assessed by acetylcholine-induced vasodilatation (10^−6^ M). Segments showing no relaxation in response to acetylcholine were considered to be endothelium-denuded. Following the application of 10^−6^ M acetylcholine, tissues were rinsed three times with Krebs solution to restore basal tension.

### Drugs

All drugs were purchased from Sigma-Aldrich Co. (USA), except rosuvastatin, which was a gift from Laboratories Astra-Zeneca, SA de CV (Mexico). Rosuvastatin, L-NAME, 4-AP, TEA, glibenclamide, clotrimazole, and cycloheximide were dissolved in distilled water. Solutions of 10^−5^ M ODQ, 10^−4^ M KT 5823, 10^−5^ M apamin plus 10^−5^ M charybdotoxin, and 10^−3^ M indomethacin were prepared using 1.39 M dimethyl sulfoxide, 1.01 M ethyl acetate, 1.73 M acetic acid, and 9.4×10^−3^ M sodium bicarbonate, respectively. Fresh solutions were made for each experiment.

### Experimental protocol

Two sets of experiments were conducted to establish the mechanism involved in the acetylcholine-induced relaxant effect on phenylephrine-precontracted rat aortic rings from rats on the standard diet, CAF-diet, or CAF-diet plus rosuvastatin.

#### First set of experiments

Thirty minutes after restoration of basal tension, 10^−6^ M phenylephrine was added to endothelium-intact aortic rings from each of the three groups of rats. Twenty minutes later, the contraction plateaued. Thirty minutes after adding phenylephrine, acetylcholine (10^−9^–10^−5^ M) began to be cumulatively added to the rings at intervals of around 5 min. Tension is reported as a percentage of the phenylephrine-induced contraction in endothelium-intact rat aortic rings (1.64±0.31 g, 100% for the standard-diet; 2.79±0.40 g, 100% for the CAF-diet; and 1.71±0.20, 100% for the CAF-diet plus rosuvastatin).

#### Second set of experiments

Aortic rings were preincubated with an inhibitor/blocker of vasorelaxation for 30 min, followed by the addition of phenylephrine. The acetylcholine-induced vasorelaxant response was evaluated 30 min later. The inhibitors/blockers included the vehicle (distilled water), 10^−5^ M L-NAME, 10^−7^ ODQ, 10^−6^ M KT 5823, 10^−3^ M 4-AP, 10^−2^ M TEA, 10^−7^ M apamin plus 10^−7^ M charybdotoxin, 10^−7^ M glibenclamide, 10^−5^ M indomethacin, or 10^−5^ M cycloheximide. After preincubation and phenylephrine pre-contraction, increasing concentrations of acetylcholine were added at intervals of about 5 min.

### Statistical analysis

Data are reported as means±SE. In all experiments, n equals the number of animals from which aortic segments were obtained (6 in each case). The effect produced by pretreatment with rosuvastatin, the specific concentration of acetylcholine, and the interaction of these two factors was examined using a three-way analysis of variance. Moreover, the influence of inhibitors/blockers on the acetylcholine-induced relaxation of aortic rings (in all three groups of rats) was determined using repeated measures two-way analysis of variance (RM-ANOVA). In each case, ANOVA was followed by the Student-Newman-Keuls *post hoc* test, performed with the SigmaPlot 12 program (Systat Software Inc., USA). Statistical significance was considered to be P<0.05 (6).

## Results

The values of the metabolic parameters (body weight, serum insulin, glucose, total cholesterol, triglycerides, and mean blood pressure) were determined for Wistar rats subsequent to a 16-week regimen based on the standard diet, the CAF-diet, or the CAF-diet plus rosuvastatin administered orally (10 mg/kg) once per day. The values of the metabolic parameters were significantly higher in rats subjected to the CAF-diet than in animals fed the standard diet or CAF-diet plus rosuvastatin (Table 1).

**Table 1:** Metabolic parameters of three groups of male Wistar rats on a 16-week regimen, including the standard diet, the cafeteria-style (CAF)-diet, and the CAF-diet plus rosuvastatin treatment.

	Standard diet	CAF-diet	CAF-diet plus rosuvastatin
Body weight (g)	413.0±3.5	454±4.0*	412.2±4.2**
Serum insulin (μIU/mL)	2.0±0.0	17.40±0.9*	11.2±0.94**
Glucose (mg/dL)	80.0±4.2	110.0±1.1*	96.0±0.05**
Total cholesterol (mg/dL)	66.99±1.0	129.69±12.5*	42.0±1.9**
Triglycerides (mg/dL)	87.66±6.2	322.74±18.43*	100.33±5.5**
Mean blood pressure (mmHg)	118.8±0.1	209.8±5.08*	146.1±4.25**

Data are reported as means±SE for 6 observations (n=6). *P<0.05 *vs* the standard diet. **P<0.05 *vs* the CAF-diet (*t*-test).

### Effect of acetylcholine on phenylephrine-precontracted rat aortic rings

**Figure 1:**
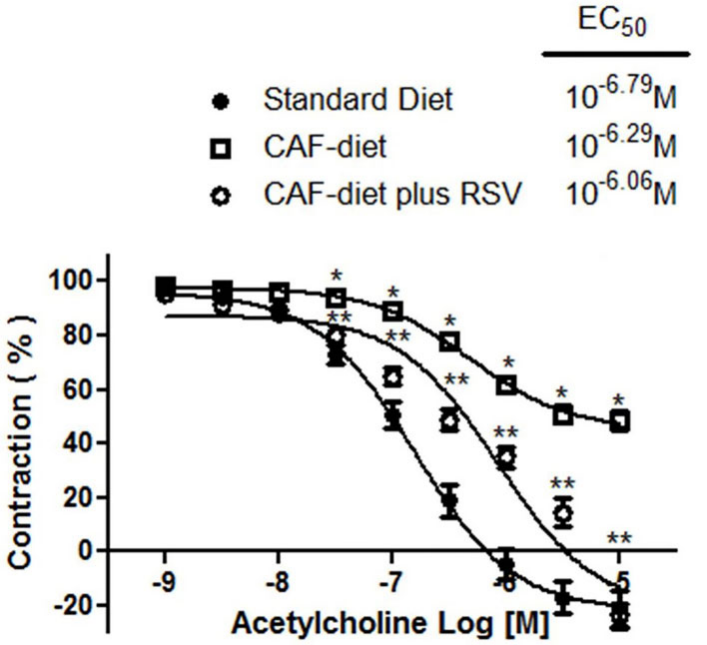
Concentration-dependent relaxation induced by 10^−9^–10^−5^ M acetylcholine on endothelium-intact rat aortic rings precontracted with 10^−6^ M phenylephrine. The rings were extracted from rats fed the standard diet, the cafeteria-style (CAF) diet, and the CAF-diet plus rosuvastatin (RSV) treatment. Data are reported as means±SE of n*=*6 observations. *P<0.05 *vs* the standard diet; **P<0.05 *vs* the CAF-diet (ANOVA).

For all three groups, 10^−9^–10^−5^ M acetylcholine caused concentration-dependent relaxation of the phenylephrine-precontracted aortic rings with intact endothelium (Figure 1). The maximum relaxation of aortic rings was 117.8±3.90% for the standard diet, 52.64±1.99% for the CAF-diet, and 123.8±4.64% for the CAF-diet plus rosuvastatin.

### Effect of L-NAME on the acetylcholine-induced relaxant response

**Figure 2:**
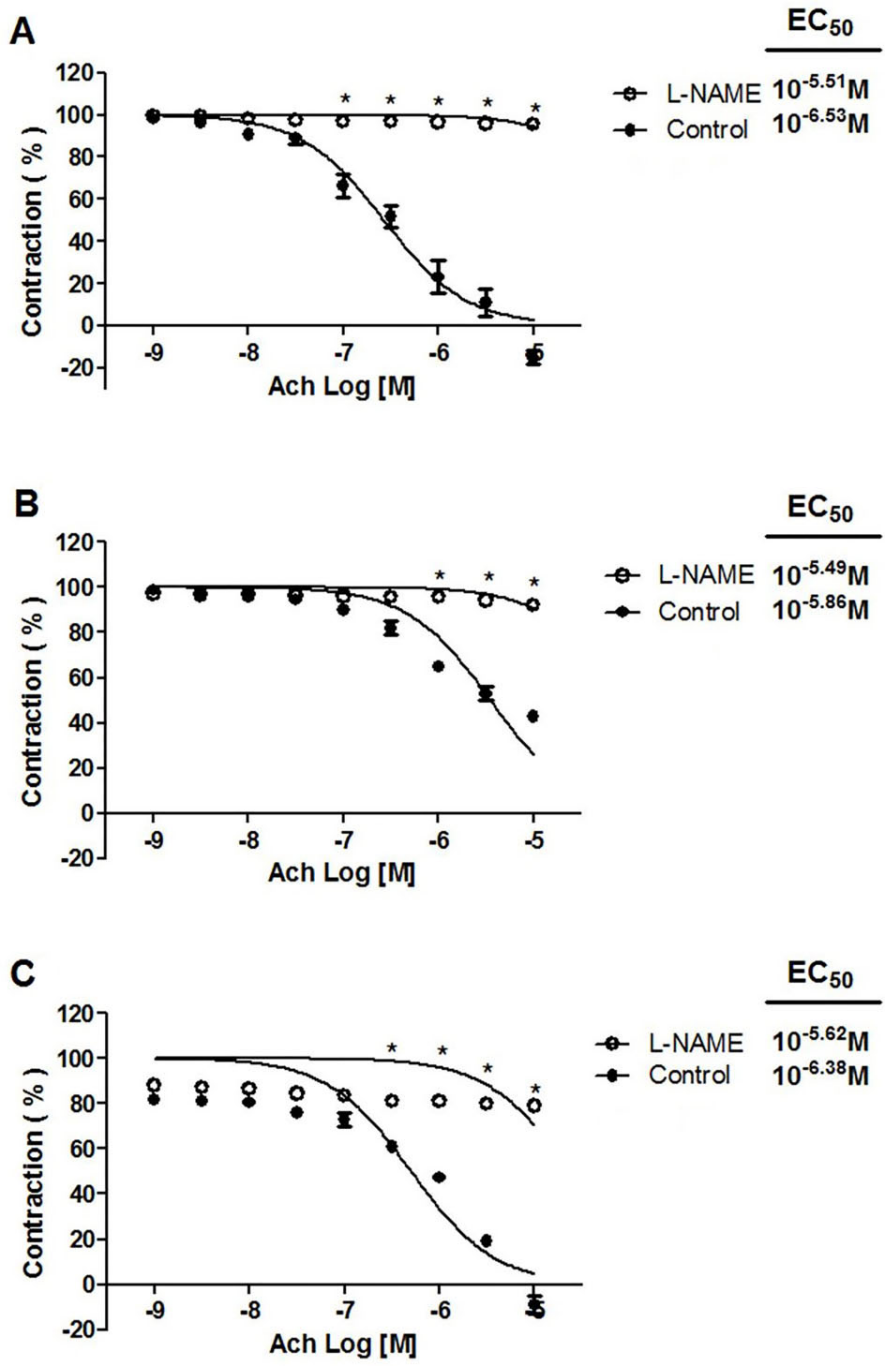
Effect of L-NAME (10^−5^ M) on acetylcholine (Ach) relaxation of endothelium-intact rat aortic rings precontracted with 10^−6^ M phenylephrine. The rings were extracted from rats fed: **A**) the standard diet; **B**) the cafeteria-style (CAF)-diet; and **C**) the CAF-diet plus rosuvastatin (RSV) treatment. Data are reported as means±SE of n=6 observations. *P<0.001 *vs* control (two-way ANOVA).

Various inhibitors/blockers were tested in relation to the acetylcholine-induced relaxation of phenylephrine-precontracted rat aortic rings. The maximum vasorelaxant effect produced by acetylcholine for the presence of 10^−5^ L-NAME in all three groups (Figure 2), as evidenced by the following data (in the absence *vs* presence of the compound): a) 110.80±0.07% *vs* 4.40±0.06% for the standard diet; b) 57.28±0.03% *vs* 7.79±0.09% for the CAF-diet; and c) 108.90±0.07% *vs* 21.16±0.10% for the CAF-diet plus rosuvastatin.

### Effect of ODQ or KT 5823 on acetylcholine-induced vasorelaxation

**Figure 3:**
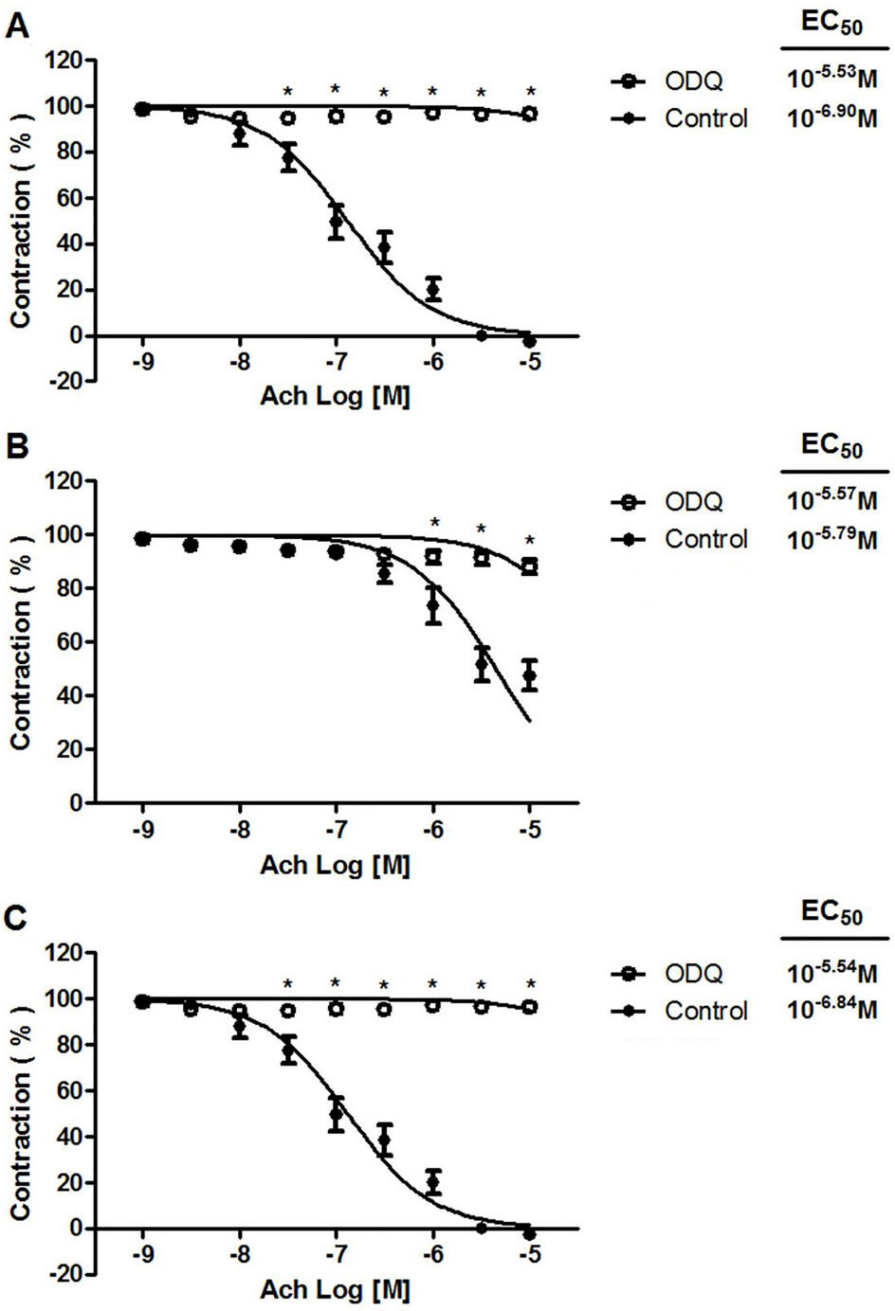
Effect of 10^−7^ M 1H-(1,2,4) oxadiazolo [4,3-a] quinoxalin-1-one (ODQ) on acetylcholine (Ach) relaxation of endothelium-intact rat aortic rings precontracted with 10^−6^ M phenylephrine. The rings were extracted from rats fed: **A**) the standard diet; **B**) the cafeteria-style (CAF) diet; and **C**) the CAF-diet plus rosuvastatin treatment. Data are reported as means±SE of n*=*6 observations. *P<0.001 *vs* control (two-way ANOVA).

**Figure 4:**
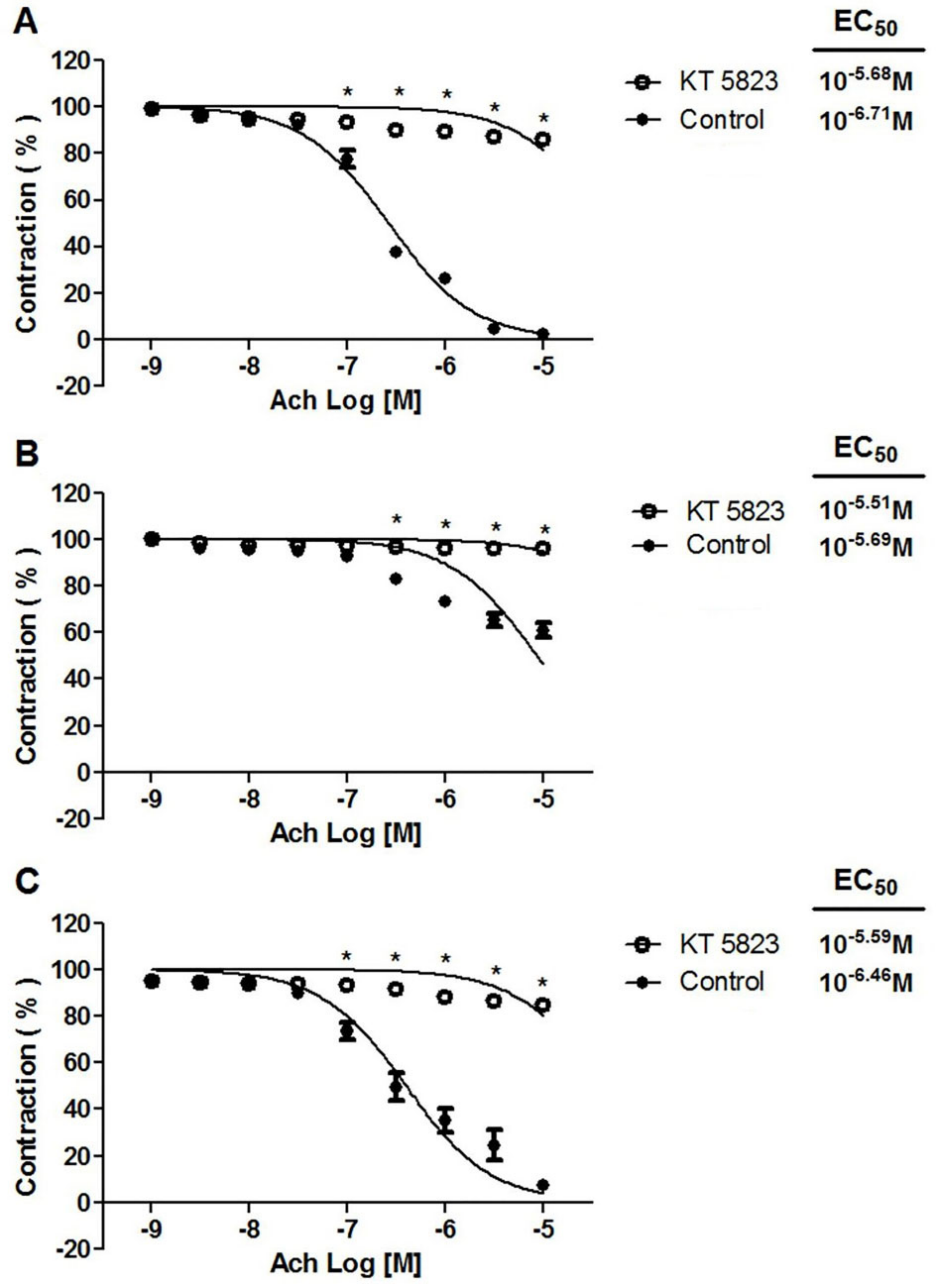
Effect of 10^−6^ M (9S,10R,12R)-2,3,9,10,11,12-hexahydro-10-methoxy-2,9-dimethyl-1-oxo-9,12-epoxy-1H-diindolo[1,2,3-fg:3',2',1'-kl] pyrrolo[3,4-i] (1,6) benzodiazocine-10-carboxylic acid, methyl ester (KT 5823) on acetylcholine (Ach) relaxation of endothelium-intact rat aortic rings precontracted with 10^-6^ M phenylephrine. The rings were extracted from rats fed: **A**) the standard diet; **B**) the cafeteria-style (CAF) diet; and **C**) the CAF-diet plus rosuvastatin treatment. Data are reported as means±SE of n=6 observations. *P<0.001 *vs* control (two-way ANOVA).

Regarding the maximum vasorelaxation generated by acetylcholine on phenylephrine-precontracted rat aortic rings (Figure 3), a significant difference (P<0.05) was found comparing the absence versus presence of 10^−7^ M ODQ pretreatment: a) 102.63±0.05% *vs* 3.64±0.02% for the standard diet; b) 52.44±0.08% *vs* 11.93±0.04% for the CAF-diet; and c) 98.50±0.06% *vs* 22.18±0.14% for the CAF-diet plus rosuvastatin. Likewise, pretreatment with 10^−6^ M KT 5823 inhibited the vasodilator response to acetylcholine in all three groups (Figure 4). The maximum vasorelaxant effects produced in the absence versus presence of KT 5823 were a) 97.77±0.08% *vs* 14.10±0.06% for the standard diet; b) 40.23±0.02% *vs* 4.19±0.04% for the CAF-diet; and c) 92.47±0.06% *vs* 15.09±0.07% for the CAF-diet plus rosuvastatin.

### Effect of 4-AP, TEA, apamin plus charybdotoxin, or glibenclamide on the acetylcholine-induced relaxant response

**Figure 5:**
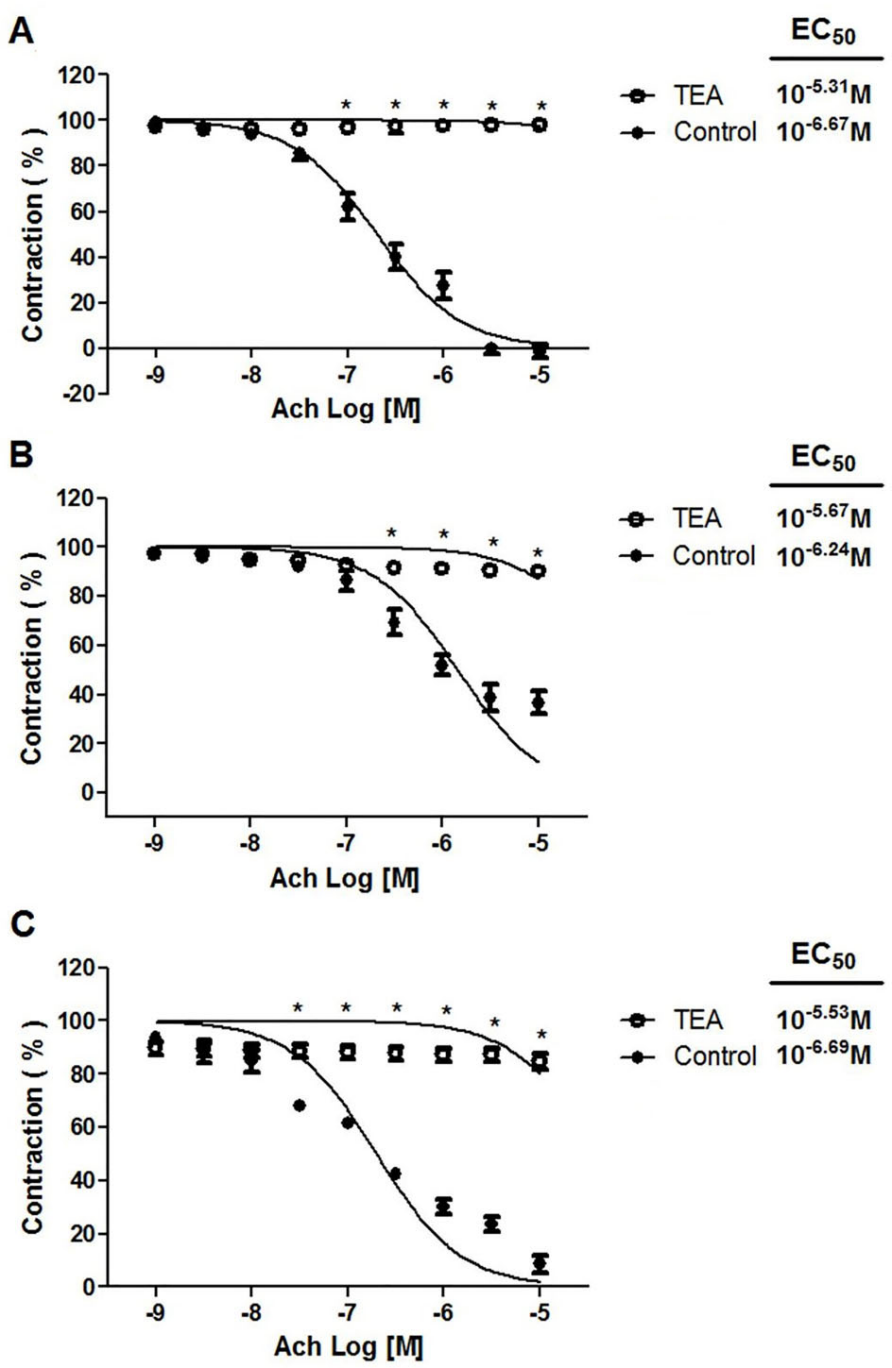
Effect of tetraethylammonium (TEA, 10^−2^ M) on acetylcholine (Ach) relaxation of endothelium-intact rat aortic rings precontracted with 10^−6^ M phenylephrine. The rings were extracted from rats fed: **A**) the standard diet; **B**) the cafeteria-style diet (CAF-diet); and **C**) the CAF-diet plus rosuvastatin treatment. Data are reported as means±SEM of n*=*6 observations. *P<0.001 *vs* control (two-way ANOVA).

**Figure 6:**
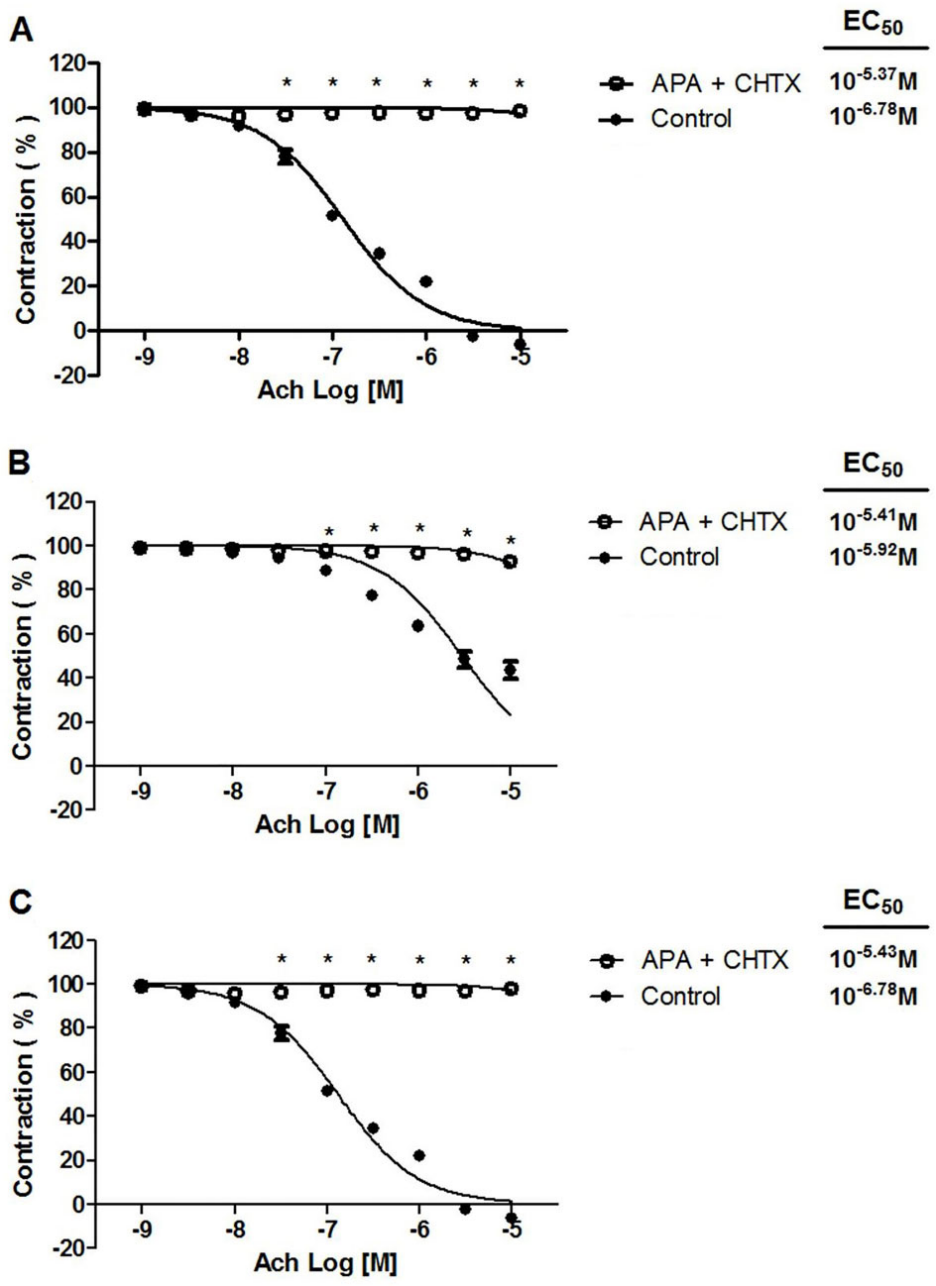
Effect of 10^−7^ M apamin plus 10^−7^ M charybdotoxin (APA + CHTX) on acetylcholine (Ach) relaxation of endothelium-intact rat aortic rings precontracted with 10^−6^ M phenylephrine. The rings were extracted from rats fed: **A**) the standard diet; **B**) the cafeteria-style diet (CAF) diet; and **C**) the CAF-diet plus rosuvastatin treatment. Data are reported as means±SE of n*=*6 observations. *P<0.001 *vs* control (two-way ANOVA).

The maximum vasorelaxant effect of acetylcholine was unaffected by 4-AP or glibenclamide (data not shown), but was influenced by 10^−2^ M TEA (Figure 5) and 10^−7^ M apamin plus 10^−7^ M charybdotoxin (Figure 6). In the absence versus presence of TEA, there was: a) 115.82±0.10% *vs* 3.80±0.05% for the standard diet; b) 63.20±0.06% *vs* 9.75±0.08% for the CAF-diet; and c) 84.80±0.09% *vs* 8.62±0.11% for the CAF-diet plus rosuvastatin, respectively. The 10^−7^ M apamin plus 10^−7^ M charybdotoxin significantly attenuated the maximum vasorelaxation induced by acetylcholine on aortic rings, as revealed by the results (in the absence *vs* presence of these compounds): a) 102.50±0.07% *vs* 4.55±0.07% for the standard diet; b) 56.49±0.05% *vs* 7.13±0.04% for the CAF-diet; and c) 96.50±0.01% *vs* 26.77±0.08% for the CAF-diet plus rosuvastatin.

### Effect of indomethacin, clotrimazole, or cycloheximide on the acetylcholine-induced relaxant response

**Figure 7:**
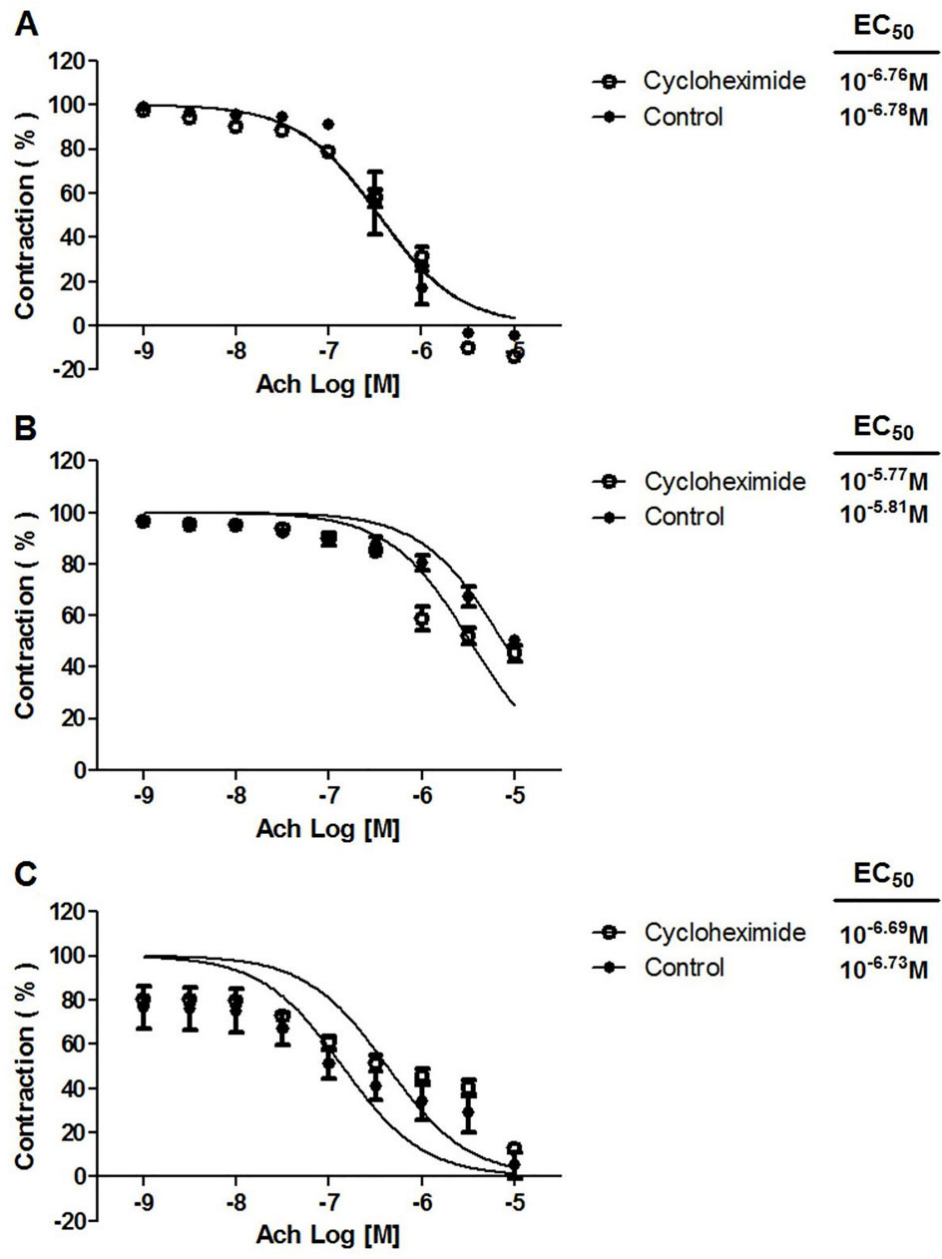
Effect of 10^−5^ M cycloheximide on acetylcholine (Ach) relaxation of endothelium-intact rat aortic rings precontracted with 10^−6^ M phenylephrine (PE). The rings were extracted from rats fed: **A**) the standard diet; **B**) the cafeteria-style (CAF)-diet; and **C**) the CAF-diet plus rosuvastatin treatment. Data are reported as means±SE of n*=*6 observations. P>0.05 *vs* control (two-way ANOVA).

The vasodilator response to acetylcholine was not modified by treatment with 10^−5^ M indomethacin or 10^−5^ M clotrimazole (data not shown) or 10^−5^ M cycloheximide (Figure 7). In the absence/presence of cycloheximide, the relaxation of the rings was: a) 108.64±3.57% *vs* 113.96±3.64% for rats fed the standard diet; b) 49.53±0.10% *vs* 51.23±1.24% for animals given the CAF-diet; and c) 95.95±6.23% *vs* 98.00±3.31%, respectively, for those given the CAF-diet plus rosuvastatin.

## Discussion

The CAF-diet is an experimental model commonly used to investigate the effect of a Western dietary regime on animal health because it represents the food intake of people in modern Western societies (7,8). The CAF-diet is a robust experimental model of metabolic syndrome that is capable of inducing obesity, glucose intolerance, and inflammation in rats (7,9), and is consistent with the results of our experiments, which showed that the 16-week CAF-diet significantly increased (P<0.05) body weight, serum insulin, glucose, total cholesterol, and triglycerides in Wistar rats (Table 1). These findings reinforced previous studies in which an 8-week CAF-diet increased the same metabolic parameters in Wistar rats (4) and are in line with previous reports linking a high-fat diet to increases in levels of glucose, cholesterol, triglycerides, and insulin (10,11).

On the other hand, the co-administration of rosuvastatin in rats subjected to the CAF-diet decreased all the parameters of metabolic syndrome (Table 1). The body weight-lowering effect produced by rosuvastatin in rats fed the CAF-diet compared to rats that were only fed the CAF-diet reinforced recent studies that suggested that treatment with rosuvastatin for 6 weeks produces a decreasing tendency in mean body weight of mice fed a high-fat diet. In these studies, the treatment with rosuvastatin decreased the weight of fatty tissue samples and significantly decreased the fat storage in adipose tissue samples (10). The body weight-lowering effect produced by rosuvastatin could be involved with a decrease in plasma levels of leptin, as previously report for pravastatin (12). In those studies, Yu et al. (12) found that pravastatin significantly decreases (P<0.05) the progressive elevation of plasma leptin and, subsequently, significantly decreases (P<0.05) the body weight and food intake in a model of rats spontaneously developing type II diabetes mellitus, the Otsuka Long-Evans Tokushima Fatty rats. Certainly, we have no clear-cut explanation about the mechanisms involved in the body weight-lowering effect produced by rosuvastatin and the above suggestion is a mere speculation.

Concerning glucose levels and insulin resistance, rosuvastatin markedly decreased hyperglycemia and hyperinsulinemia developed as part of the metabolic syndrome. These effects could stem from several well-documented mechanisms of statins: a) the preservation of pancreatic beta cell function due to an increase in pancreatic proliferation (13); b) greater insulin sensitivity (14,15); c) elevated basal translocation of GLUT-4 (9); and d) reduced insulin content together with enhanced insulin secretion. Salunkhe et al. (14) added rosuvastatin (0.2 mg/day) to the drinking water of rats subjected to a high fat diet, resulting in a decrease in plasma glucose fostered by an increase in insulin sensitivity. This outcome may account for the lower hyperinsulinemia and hyperglycemia observed presently with the CAF-diet plus rosuvastatin treatment (compared to the CAF-diet alone).

The drop in cholesterol and triglyceride levels detected herein can also be explained by some well-recognized mechanisms of statins. Statins lower cholesterol and triglyceride levels due to their inhibitory action on 3-hydroxy-3-methyl glutaryl coenzyme A reductase, the limiting enzyme for cholesterol synthesis (16). Statins are known to bind at nanomolar concentrations, displacing the natural substrate (HMG-CoA), which binds at micromolar concentrations (17). It was described that statins do not only compete with the natural substrate for the active site of the enzyme, but also alter the conformation of the enzyme and limit its functional activity, thus improving the efficiency and specificity of these drugs (18).

The rosuvastatin-induced decrease in blood pressure is possibly caused by its pleiotropic actions on nitric oxide (NO), including: a) the inhibition of the production of mevalonate, which negatively regulates the expression of eNOS (19); b) a boost in eNOS activity (20) through the post-translational activation of phosphatidylinositol 3-kinase and protein kinase Akt, and/or interaction with the chaperone HSP90 (causing greater phosphorylation of Akt and eNOS) (19); and c) the reduction in intra-platelet nitrotyrosine, a compound that generates reactive oxygen species (21).

### Effect of acetylcholine on phenylephrine-precontracted rat aortic rings

Acetylcholine produces an endothelium-dependent relaxation in vascular smooth muscle preparations that are precontracted with high concentrations of potassium, noradrenaline, and other vasoactive agents (22). Several reports have shown that atropine-sensitive muscarinic receptors are involved in this vasorelaxant response (23,24). In the present investigation, the aortic rings were precontracted with phenylephrine and left to reach a stable plateau. Subsequently, a concentration-response curve to acetylcholine (10^-9^-10^-5^ M) showed vasodilator responses of distinct magnitudes for the three groups. The vasorelaxant responses were 117.8±3.90% for the control rats, 52.64±1.99% for animals with metabolic syndrome, and 123.8±4.64% for those with metabolic syndrome plus rosuvastatin treatment. The vasorelaxant responses to acetylcholine in phenylephrine-precontracted aortic rings of rats fed with the CAF-diet were significantly lower compared to those in rats fed with the standard diet. Treatment with rosuvastatin significantly reversed the decrease in the vasorelaxant responses to acetylcholine in phenylephrine-precontracted aortic rings of rats fed with the CAF-diet. These results are consistent with previous findings in which: 1) acetylcholine-mediated vascular relaxation is decreased in the epineural arterioles of the sciatic nerve of obese Zucker rats at 32 weeks of age (a model of metabolic syndrome) compared to lean rats; and 2) the treatment with rosuvastatin for 12 weeks significantly reversed those decreases in acetylcholine-mediated vascular relaxation. Oltman et al. (25) suggest that superoxide and nitrotyrosine levels in epineural arterioles and superoxide levels in the aorta were increased in obese Zucker rats. In epineural arterioles the increase in superoxide appears throughout the vessel wall, whereas the increase in nitrotyrosine staining occurs predominantly in the endothelial layer. Moreover, the authors suggest that the levels of superoxide and nitrotyrosine were significantly decreased when obese Zucker rats were treated with rosuvastatin. In addition, vasorelaxant responses obtained in the group of animals with metabolic syndrome plus rosuvastatin treatment could also be attributed to the ability of statins to increase the synthesis of NO through several mechanisms. For example, the drug inhibits the production of mevalonate, an isoprenoid intermediate that prevents the isoprenylation of Rho GTPase, which in turn negatively regulates eNOS expression (19,26). Additionally, rosuvastatin is able to intensify eNOS activity (27,28) by the post-translational activation of phosphatidylinositol 3-kinase and/or protein kinase Akt, as well as reduce caveolin-1 activity (29).

### Mediators involved in the vasodilatory effect of acetylcholine

Acetylcholine induces vasodilatation in different vascular beds by stimulating the release of relaxant factors derived from endothelium such as NO (22). The fact that L-NAME (a direct inhibitor of NOS) significantly (P<0.05) attenuated the vasorelaxant responses to acetylcholine in aortic rings of animals fed the standard diet, animals fed the CAF-diet, and animals fed the CAF-diet and treated with rosuvastatin suggest the involvement eNOS in all three groups.

Several reports (22–24) suggest that the vasodilation caused by NO is mediated by the stimulation of guanylate cyclase, an enzyme responsible for the production of cGMP and the subsequent activation of protein kinase G. The present treatment of aortic rings with ODQ (an inhibitor of soluble guanylyl cyclase enzyme) or KT 5823 (an inhibitor of protein kinase G) inhibited the relaxant response to acetylcholine. Thus, cGMP and protein kinase G seem to be involved in acetylcholine-induced vasodilation.

Since the relaxant response to acetylcholine was inhibited by the presence of tetraethylammonium, potassium channels participated in the vasodilator effect. There is considerable evidence that the opening of potassium channels in vascular smooth muscle induces hyperpolarization and consequently dilates the arteries (30). Smooth muscle tissue has a variety of potassium channels, such as Ca^2+^-activated, voltage-dependent, and ATP-sensitive K^+^ channels (31). The relaxation herein induced by acetylcholine was significantly attenuated by TEA (a Ca^2+^-activated K^+^ channel blocker and non-specific voltage-activated K^+^ channel blocker) as well as apamin plus charybdotoxin (blockers of small- and large-conductance Ca^2+^-activated K^+^ channels, respectively), but was not affected by the presence of 4-AP (a voltage-activated K^+^ channel blocker) or glibenclamide (an ATP-sensitive K^+^ channel blocker). Hence, K^+^ channels activated by Ca^2+^ appear to contribute to acetylcholine-induced vasodilation.

Prostaglandins and endothelium-derived hyperpolarizing factor are important mediators of vasodilation (32,33). However, these mediators did not contribute to the acetylcholine-mediated vasorelaxant effects observed in the current investigation, since indomethacin or clotrimazole did not modify those effects.

Finally, cycloheximide, a general protein synthesis inhibitor, did not modify the vasorelaxation produced by the acute application of acetylcholine on aortic rings of animals fed the standard diet, animals fed the CAF-diet, and animals fed the CAF-diet and treated with rosuvastatin (Figure 7), which excluded the possible involvement of protein synthesis in the vasorelaxant effect produced by this vasodilator agent. The above results contrasted with previous findings in which preincubation with cycloheximide reduced the vasodilator responses produced by the acute application of the methyl ester of rosuvastatin (34) and rosuvastatin (4) on aortic rings of rats fed the standard diet or after the 8-week CAF-diet, respectively. The lack of the effect of cycloheximide on the vasorelaxant response to acetylcholine can be explained by its immediate vasodilator effect being dependent on direct stimulation of muscarinic receptors (24), while the vasorelaxant response to rosuvastatin involves stimulation of NOS (4).

In conclusion, the current study showed that the subchronic administration of rosuvastatin to rats fed the hypercaloric diet lowered the parameters that indicate metabolic syndrome. Moreover, this treatment normalized the vasodilator response caused by acetylcholine, the latter involving the NO/cGMP/PKG/Ca^2+^-activated K^+^ channel pathway in the vasorelaxant effect observed.
